# Population Genetic Analysis of the Estonian Native Horse Suggests Diverse and Distinct Genetics, Ancient Origin and Contribution from Unique Patrilines

**DOI:** 10.3390/genes10080629

**Published:** 2019-08-20

**Authors:** Caitlin Castaneda, Rytis Juras, Anas Khanshour, Ingrid Randlaht, Barbara Wallner, Doris Rigler, Gabriella Lindgren, Terje Raudsepp, E. Gus Cothran

**Affiliations:** 1 College of Veterinary Medicine and Biomedical Sciences, Texas A&M University, College Station, TX 77843, USA; 2 Sarah M. and Charles E. Seay Center for Musculoskeletal Research, Texas Scottish Rite Hospital for Children, Dallas, TX 75219, USA; 3 Estonian Native Horse Conservation Society, 93814 Kuressaare, Saaremaa, Estonia; 4 Institute of Animal Breeding and Genetics, University of Veterinary Medicine Vienna, 1210 Vienna, Austria; 5 Department of Animal Breeding and Genetics, Swedish University of Agricultural Sciences, 75007 Uppsala, Sweden; 6 Livestock Genetics, Department of Biosystems, KU Leuven, B-3001 Leuven, Belgium

**Keywords:** Estonian Native Horse, autosomal STRs, MSY haplotypes, mtDNA D-loop, *DMRT3*, gait, genetic diversity, ancestry

## Abstract

The Estonian Native Horse (ENH) is a medium-size pony found mainly in the western islands of Estonia and is well-adapted to the harsh northern climate and poor pastures. The ancestry of the ENH is debated, including alleged claims about direct descendance from the extinct Tarpan. Here we conducted a detailed analysis of the genetic makeup and relationships of the ENH based on the genotypes of 15 autosomal short tandem repeats (STRs), 18 Y chromosomal single nucleotide polymorphisms (SNPs), mitochondrial D-loop sequence and lateral gait allele in *DMRT3*. The study encompassed 2890 horses of 61 breeds, including 33 ENHs. We show that the expected and observed genetic diversities of the ENH are among the highest within 52 global breeds, and the highest among 8 related Northern European ponies. The genetically closest breeds to the ENH are the Finn Horse, and the geographically more distant primitive Hucul and Konik. ENH matrilines are diverse and relate to draught and Pontic-Caspian breeds. ENH patrilines relate to draught breeds, and to a unique haplogroup not described before. None of the 33 ENHs carried the “gait” mutation, but the mutation was found in 2 Huculs. The study demonstrates that the ENH is a genetically distinct and diverse breed of ancient origin with no notable pressure of selective breeding.

## 1. Introduction

Archeological findings indicate that wild horses lived on the territory of present-day Estonia as early as ~10,000–9000 years before present (BP) but went extinct and did not contribute to horse domestication. Instead, horses domesticated elsewhere arrived in Estonia with human migration from the East during the Late Iron Age/Early Bronze Age ~3000 years BP [1,2,3]. The first written sources mentioning horses in Estonia (Livonia) were those by Adam of Bremen, a German Medieval chronicler and traveler of the 11th century, and by the Livonian Chronicle of Henry from the 13th century [4,5]. Thus, the origin of the Estonian Native Horse (ENH) dates back thousands of years and remains vague, though phenotypic characteristics of the ENH favor alleged claims that it is one of the few surviving descendants of the forest horse, Tarpan (*Equus ferus*), which populated Eastern Europe a few centuries ago and was declared extinct only in 1909 [5,6,7,8].

The geographic location of Estonia between Eastern and Western powers and its turbulent political history of being conquered and ruled by many has also shaped horse populations. For example, local stallions and mares were frequently deported to neighboring territories: to Sweden as military horses and to Russia to improve their local horse breeds. In the 19th century, driven by agricultural needs, native horses were crossed with larger breeds, such as the Hackney and Ardennes, to increase body size and improve performance. These breeding efforts, along with the introgression of the Arabian and Finn Horse, created the Estonian Heavy Draught and Tori segments of Estonian horse populations [5,8,9]. The original ENH primarily remained isolated on the western islands of Estonia and organized breeding started in 1921 when the Estonian Native Horse Breeders Society was founded and a studbook established [5]. The current population size of the ENH is 2519 individuals [10] and the breed is listed among endangered-maintained domestic animal breeds by the World Food and Agriculture Organization [11]. Today, only pure breeding is permitted for the ENH [9], and the breed is locally protected and maintained by the Estonian Native Horse Conservation Society [5].

The Estonian Native Horse has an average wither height of 146 cm and average weight of 430 kg, placing it phenotypically with what is commonly considered ponies [11]. The breed is well adapted to the local climate and environment and does not need additional fodder or a proper stable during the snow-free time of the year. The breed is also known for disease resistance, efficient feed use, endurance and good temperament. At present, the ENH is mainly used for sports and recreation involving harness and saddle riding and equestrian tourism [5].

Despite the long cultural history, overall popularity and endangered-maintained status, very little is known about the genetic makeup and origin of ENH. A few layman articles have documented the rich variety of coat color patterns of the ENH [12,13], which is a clear indication of genetic diversity. The breed contributed to the recent discovery of the molecular basis of the Dun color [14], and according to anecdotal claims, may exhibit lateral gait. The only population genetic study that has involved the ENH was published recently and compared the genetic diversity, population structure and genetic affinity of 18 geographically/historically related breeds, including the ENH, Tori and Estonian Heavy Draught [9]. An intriguing finding of this study was that there is a shared gene pool between the ENH and Yakutian and Mongolian horses, posing additional questions about the ancestry of this native breed.

The aim of the present study is to further investigate the genetic makeup, relationships and ancestry of the ENH. Our analysis is based on autosomal short tandem repeats (STRs), Y chromosome single nucleotide polymorphisms (SNPs), mitochondrial DNA (mtDNA) D-loop region, and the *DMRT3* “gait” mutation. The study encompasses a global collection of 61 horse breeds, including several native ponies and primitive breeds.

## 2. Materials and Methods

### 2.1. Ethics Statement

Procurement of hair samples followed the United States Government Principles for the Utilization and Care of Vertebrate Animals Used in Testing, Research and Training. These protocols were approved as AUP # 2018-0342 CA at Texas A&M University, TX, USA.

### 2.2. Animals for Comparative Analysis

Hair samples were obtained from 33 Estonian Native horses and 25 Finn Horses—a geographically and historically related breed to the ENH. All horses were registered by respective breed societies. In addition, we obtained genotyping data for 15 genome-wide STRs for 2617 horses of 50 breeds from the archives of the Animal Genetics Laboratory, Texas A&M University. For mitochondrial comparisons we obtained 215 sequences from the National Center for Biotechnology Information (NCBI) database representing 30 European horse breeds, 9 of these breeds were utilized only for mitochondrial analysis. Thus, a total of 2890 horses of 61 breeds were analyzed in this study. Horse breeds were chosen to provide a collective representation of modern domestic horse breeds with respect to geographical location and diversity. List of breeds and the number of horses per breed are documented in Table S1.

### 2.3. DNA Isolation and STR Genotyping

Genomic DNA was extracted from hair follicles of 58 horses (33 ENH and 25 Finn Horses) using the PUREGENE^®^ DNA purification kit following the manufacturer’s protocol (QIAGEN, Germantown, MD, USA). Individuals were genotyped for 15 STR markers (AHT4, AHT5, ASB2, ASB17, ASB23, HMS6, HTG6, HMS7, HMS3, HMS2, HTG4, VHL20, HTG7 and HTG10 and LEX33) for population analysis and added to the genotype depository of the Animal Genetics Laboratory, Texas A&M University. Information about the genomic location of the STR loci, primer sequences and allele sizes have been previously published [15]. STR genotyping was performed using an ABI PRISM 377 (Applied Biosystems, Foster City, CA, USA) following previously described methods [16]. 

### 2.4. Population Diversity and Structure Analysis

Each locus was tested in each population for deviation from Hardy‒Weinberg equilibrium (HWE) using the Chi-square test imbedded in GENEALEX 6.5 [17]. The STR relationship between the 33 ENH and a global cohort consisting of 2643 individuals from 51 additional breeds was determined by the majority-rule consensus of restricted maximum likelihood (RML) trees (Figure S1). The consensus tree was generated using the Reynolds’ [18] chord distance and based upon 1000 bootstrapped allele frequency datasets using the PHYLIP 3.69 package [19]. Trees were visualized by MEGA6 and SplitsTree4 [20,21]. The consensus tree established a baseline for genetic relationships among the ENH and other breeds. Based on the consensus tree and geographic location, the genetic relationship among the ENH and 22 breeds of the global cohort were mapped out using GENETIX 4.05 [22] to generate a factorial correspondence analysis (FCA). The genetic diversity for breeds in Group A was calculated using GENEALEX 6.5 [17]. Secondary analysis of the relationship between the ENH and 22 breed group was performed using Reynold’s [18] chord distances; trees were visualized using SplitsTree4 [20,21]. STRUCTURE 2.3.3 [23] was used to study admixture patterns between the ENH and 8 putative related breeds (Group A; N = 423) and the remaining 14 breeds (Group B; N = 660) using a burn-in value of 50,000 and 100,000 Markov chain Monte Carlo (MCMC) iterations. Runs for each value of K (Group A: K = 2 to K = 10; Group B: K = 2 to K = 17) were reiterated 10 times. Consistent with previous studies [15,24,25], the consensus tree identified the Exmoor Pony (N = 70) as most primitive in clade III and Group A (Figure S1). Thus, Exmoor Pony was used as an out-group for all STRUCTURE analysis instead of the Przewalski’s Horse to prevent inflation of genetic distance axes (Figure S4) between the ENH and other breeds. CLUMPP [26] and DISTRUCT [27] were used to align the iterations from STRUCTURE 2.3.3 and display grouped breed results. The most informative clusters were determined based upon ΔK values [28] extracted using the Structure Harvester application [29].

### 2.5. Y Chromosome and Mitochondrial Variant Screening and Haplotype Analysis

All ENH males (N=14) were selected for male specific region of Y (MSY) SNP screening. MSY chromosome haplogroup was determined by genotyping 18 variants (rBF, rAY, rAX, rA, rB, rT, sAL, sPZ, rW, rX, rAF, rOR, rAG, rAH, rAJ, rAI, rDS, rAE) using LGC Kasp technology as described previously [30,31]. Information about MSY markers, indicative haplogroups, ancestral and derived alleles, and flanking sequences for genotyping is presented in Table S2 [30,31]. 

PCR amplification and sequencing of the partial mtDNA D-loop in 9 unrelated ENH samples were done as described previously [32]. Sequences were analyzed in the Molecular Evolutionary Genetics Analysis (MEGA) tool [20] using the horse mtDNA sequence X79547 as a reference. We also included 215 D-loop sequences representing 30 European horse breeds retrieved from the NCBI (Table S3) in the analysis. Haplotypes were generated using DnaSP 5.10.1 [33] and compared to the horse haplogroups defined by Achilli and colleagues [34].

### 2.6. DMRT3 Gait Genotyping

Previously published TaqMan™ SNP genotyping assays for the *DMRT3* lateral gait associated allele were utilized for gait analysis [35] in the ENH and the 8 related breeds (Group A). Of the latter, 6 breeds (Finn Horse, Konik, Gotland Pony, Icelandic Horse, Norwegian Fjord, and Shetland Pony) have already been genotyped for the *DMRT3* gait allele [36]. Therefore, we genotyped the remaining 3 breeds for the gait mutation in a total of 114 horses: 33 ENH, 52 Hucul Horses, and 29 Exmoor Ponies. We used a CFX-96 Real Time-PCR machine (Bio Rad, Hercules, CA, USA) and corresponding software for PCR amplifications, genotyping and allelic discrimination. The thermal conditions were: priming at 60 °C for 1 min, initial denaturation at 95 °C for 10 min, 40 cycles at 92 °C for 15 sec each, annealing at 60 °C for 30 s, and extension at 60 °C for 1 min, followed by a final extension at 65 °C for 10 min. The 8 µL reactions contained 0.208 µL of TaqMan™ assay, 30 ng template DNA and 4.2 µL of ABI TaqMan Universal Master mix, no uracil-N-glycosylase (UNG) (Applied Biosystems). 

## 3. Results

### 3.1. Genetic Diversity and Relationships of the Estonian Native Horse

All 15 STR loci tested were found to be polymorphic and did not deviate from HWE in the populations (*p* < 0.05). The genetic diversity (measured as observed and expected heterozygosity; HO and HE) was in the range of 0.489–0.779 (HO) and 0.521–0.787 (HE) with low standard error for the global cohort of 52 breeds, and the ENH had one of the highest levels of HO (0.766) and HE (0.723) (Table S1). Genetic relationship analysis of 52 breeds grouped the ENH within a large clade of 26 European draught and pony breeds (Figure S1, clade II) and a smaller clade with 9 Northern European pony breeds (Figure S1, clade III). Breeds in clade II had a genetic diversity range of 0.489‒0.0766 for HO and 0.521‒0.723 for HE (Table S1). Within the smaller group of 9 ponies (clade III), the ENH maintained the highest genetic diversity with HO of 0.766 and HE of 0.723, while the Sheltland Pony and Exmoor Pony had the lowest observed and expected diversity level. Next to the ENH, the Hucul horse had the second highest diversity of 0.732 (HO) and 0.709 (HE) (Table 1). 

**Figure 1:**
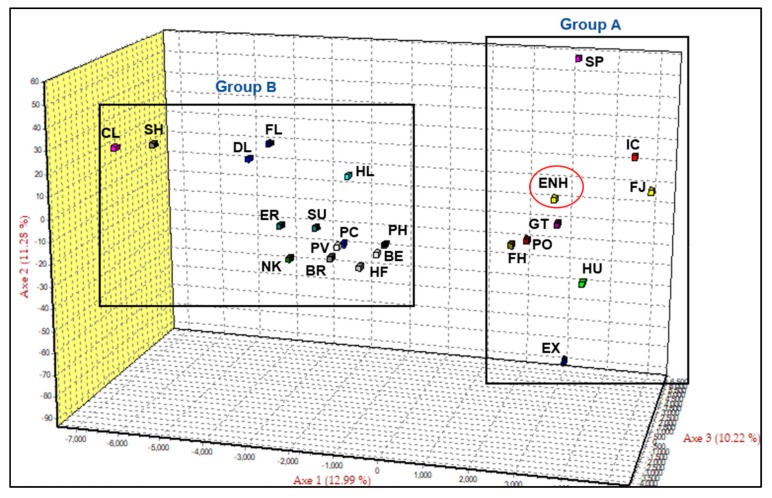
Factorial correspondence analysis (FCA) of Estonian Native Horse (ENH; circled) in comparison with 22 horse breeds. Clydesdale (CL), Shire (SH), Dales Pony (DL), Fell Pony (FL), Highland Pony (HL), Eriskay Pony (ER), Suffolk Punch (SU), Percheron (PC), Noriker (NK), Posavina (PV), Breton (BR), Polish Heavy Horse (PH), Belgian Draught (BE), Haflinger (HF), Hucul (HU), Konik (PO), Finn Horse (FH), Gotland Pony (GT), Icelandic Horse (IC), Norwegian Fjord (FJ), Shetland Pony (SP), Exmoor Pony (EX).

For further analysis we selected 22 of the 26 European draught breeds from clade II (Figure S1), applied the FCA function of GENETIX 4.05 to determine relationships with the ENH and draught breeds, and generated two distinct groups—A and B (Figure 1). Axis 1 explained the largest portion of variation (12.99%) and separated the ENH and 8 additional Northern European pony breeds (Group A) from the remaining East and West European draught breeds (Group B). Axes 2 and 3 explained 11.28% and 10.22% of the variation, respectively. Group A consisted of the ENH, Konik, Hucul, Gotland Pony, Finn Horse, Norwegian Fjord, Icelandic Horse, Shetland Pony, and Exmoor Pony. Two-dimensional analysis of axes one versus two (12.99%; 11.28%) and axes one versus three (12.99%; 10.22%) are presented for greater clarity of separation on paired axes (Figure S2).

**Figure 2:**
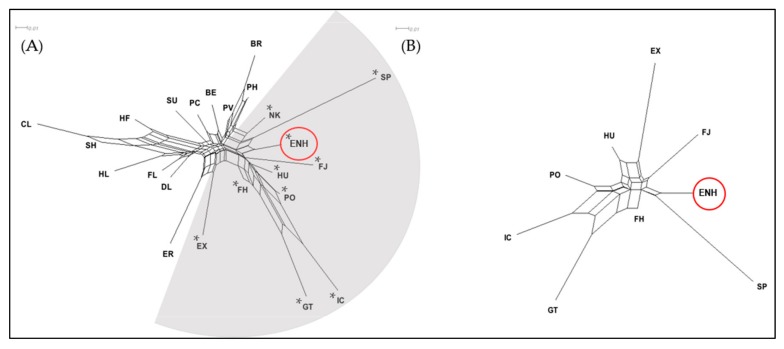
Neighbor-network trees based on Reynolds genetic distance of 23 horse breeds. (**A**) Distance mapping of the ENH (circled) and 22 breeds created two subpopulations; breeds indicated with asterisks (*) in the grey-shaded area belong to Group A in Figure 1. (**B**) Distance mapping of the ENH (circled) with 8 related breeds. Branch length indicates population divergence. Breed abbreviations are the same as in Figure 1.

The neighbor-network analysis of the 22 breeds and ENH confirmed the FCA results as the horse breeds segregated in a similar pattern to groups A and B of Figure 1 (Figure 2A). Each analysis of diversity between horse breeds placed the ENH with 8 Northern European pony breeds. Further analysis of the ENH and 8 Northern European ponies in Group A defined the Hucul, Konik, Finn Horse, and Norwegian Fjord as more closely related to the ENH based on branch length (Figure 2B). The Finn Horse had the shortest branch length and fell closest to the center of the neighbor-network tree and the Icelandic Horse, Exmoor and Shetland ponies had the longest branch lengths. 

### 3.2. Population Structure

**Figure 3:**
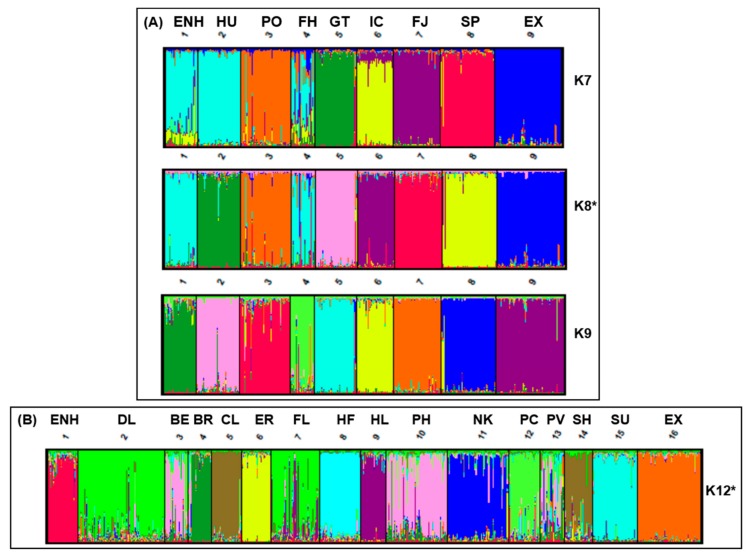
STRUCTURE analysis of the Estonian Native Horse (ENH) with (**A**) the 8 Northern European pony breeds in Group A at K = 7, K = 8 and K = 9, and (**B**) with the remaining 14 breeds in Group B and Exmoor Pony at K = 12. Optimal population clustering for Group A and B indicated by asterisks (*). Breed abbreviations are listed in Figure 1.

To further investigate the relationship between the Estonian Native Horse and putatively related breeds, we analyzed admixture patterns of the two groups determined by FCA and conducted distance mapping using STRUCTURE. Based on the consensus RML tree (Figure S1), the Exmoor Pony was most primitive for breeds in Group A and thus considered as a consistent outgroup for distance and diversity analysis between the ENH-related breeds in cluster II and cluster III. Groupings of K = 2 through K = 10 were examined for Group A and K = 2 through K = 17 for Group B with ten iterations for each K value. For Group A, the most significant ΔK was K = 8 (Figure 3A) where each population defined a unique cluster except the ENH, which clustered with the Finn Horse (light blue). At K = 7 the ENH, Hucul, and Finn Horse occupied a single cluster signifying similar ancestry. However, at K = 8 the Hucul breed separated into its own group but the ENH and Finn Horse remained together, suggesting recent admixture and strengthening of the population structure documented in a previous study [9]. Horse breeds which occupy single clusters at K = 7 (Konik, Gotland Pony, Icelandic Horse, Norwegian Fjord, Shetland Pony, and Exmoor Pony) indicate a greater genetic divergence and little admixture with the ENH. Percent contributions of each breed in Group A to a designated cluster are given in Table S4 and Figure S3, which shows changes (delta) in the LnP(D) admixture values. In contrast to Group A, population structure analysis between the ENH and the remaining 14 breeds resulted in discrete clusters at K = 12 (Figure 3B). 

### 3.3. Y Chromosome and Mitochondrial Haplogroups

**Figure 4:**
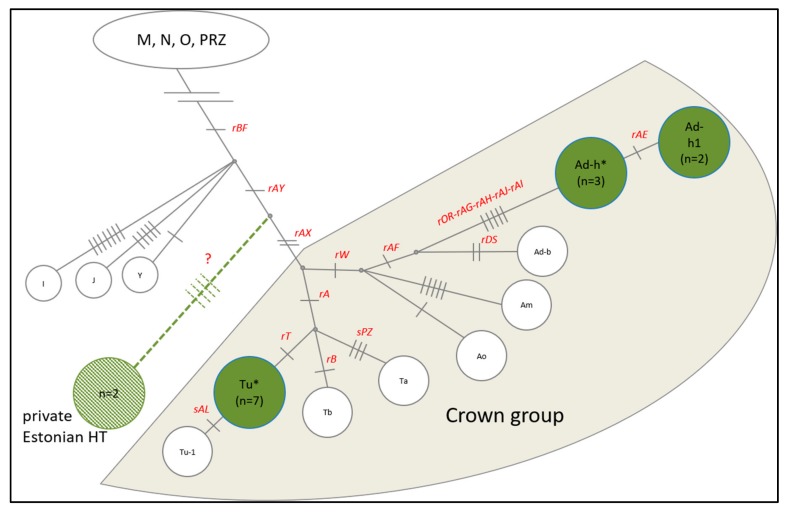
Horse Y chromosome haplogroups (HGs) based on 18 SNPs (in red font). Green circles represent the three identified HGs in the ENH; a novel HG outside the Crown group is shown in light green.

In addition to STR analyses, Y chromosome genotyping and haplogroup (HG) analysis was performed to infer the genetic history and relationships of ENH male lineages. Genotyping 18 previously described MSY SNPs [30,31] in 14 ENH males resulted in three distinct haplogroups (Figure 4). Five males grouped into the European draught horse clade (Ad-h), seven into the clade (Tu*), which was previously detected in Welsh Ponies and Franches-Montagnes [30]. Notably, two ENH males clustered outside the crown haplogroup, having the derived allele at rAY and the ancestral allele at rAX (Table S5). This allelic combination has not been observed before and represents a novel Y chromosome HG. Y chromosome genotypes of ENH males are presented in Table S5.

**Figure 5:**
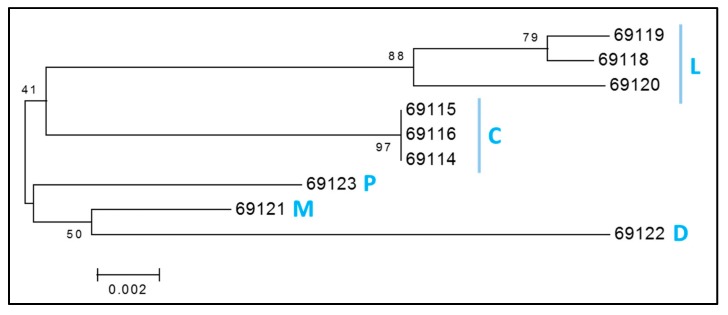
Consensus neighbor-joining tree of mitochondrial haplogroups (HGs) of 9 ENHs. Blue letters represent five identified HGs. The tree was drawn based on 1000 bootstrap replicates, values shown as percentages at branch points. HGs are denoted by letters according to previous nomenclature [34].

From a maternal perspective, we found 7 mitochondrial haplotypes in the 9 ENH genotyped (Figure 5). The haplotypes corresponded to 5 HGs; three individuals fell into HG-L, which is the most frequent HG in Europe [34] and also included 14 breeds (Asturcon, Pottoka, Vyatskaya, Konik, Haflinger, Icelandic Horse, Polish Heavy Horse, Posavina, Shire, Cartujano, Breton, Highland Pony, Shetland Pony and Clydesdale) for which mtDNA data was retrieved from NCBI (Table S3). Three other ENHs shared an identical haplotype within HG-C. Finally, 3 ENHs were assigned into 3 different haplogroups—HG-P, HG-M and HG-D. Mitochondrial diversity test statistics are documented in Table S6.

### 3.4. Genotyping for Gait Mutation in DMRT3

To determine whether ENH carries the gait mutation, we genotyped 33 ENHs for the lateral gait allele in DMRT3 [37]. In addition, DMRT3 genotypes were determined for 52 Hucul and 29 Exmoor ponies. These two breeds are related to ENH together with 6 other breeds in Group A but have not yet been genotyped for the gait allele [36]. All ENHs and Exmoor Ponies were homozygous for the wild type allele, while two Hucul individuals were heterozygous for the gait mutation (Table 2).

## 4. Discussion

Here we present a detailed analysis of the genetic makeup of the Estonian Native Horse based on the genotypes of 15 autosomal STRs, 18 Y chromosome SNPs, mitochondrial D-loop sequence and lateral gait allele in *DMRT3* [37]. To determine genetic relations of the ENH with other horse breeds (Table S1), the analysis included autosomal STR and/or mtDNA genotypes from a total of 2890 horses of 61 breeds. This is currently the most extensive and focused genetic study of this native horse breed and only the second population genetic analysis where the ENH has been included [9]. Previously, the ENH was studied together with two other Estonian breeds, the Tori Horse and Estonian Heavy Draught Horse, for diversity and relationships with 15 other breeds (total 586 horses) [9] using the same autosomal STRs as in this study. Therefore, our results are comparable to the previous study, but also expand it with regards the scope of breeds and number of animals involved. In addition, this is the first study of the ENH for Y chromosome and mitochondrial haplotypes and the gait mutation.

It is noteworthy that among the 52 horse breeds included in STR-based diversity analysis, the Estonian Native Horse had the second highest observed heterozygosity (HO) of 0.766 (Table 1 and Table S1), standing next to the Malopolski Horse (Table S1), which is a recently developed breed with high within-breed genetic diversity thanks to the contribution of multiple European warmblood breeds [38]. Overall, our HO estimates were consistent with other STR-based studies for the ENH (our 0.766 vs. 0.741) [9] and closely related pony breeds in Group A (Figure 1): Hucul (our 0.732 vs. 0.71) [39], Finn Horse (our 0.717 vs. 0.713) [9], Icelandic Horse (our 0.711 vs. 0.659) [40], Konik (our 0.696 vs. 0.669 and 0.68) [39,40], Norwegian Fjord (our 0.694 vs. 0.703 and 0.709) [9,40], Gotland Pony (our 0.650 vs. 0.63) [41], Shetland Pony (our 0.649 vs. 0.641) [40], and Exmoor Pony (our 0.619 vs. 0.606) [42]. Our HE estimates for these breeds in Group A follow similar patterns for the ENH (our 0.723 vs 0.718) [9], Hucul (our 0.709 vs. 0.72) [39], Finn Horse (our 0.705 vs. 0.722) [9], Icelandic Horse (our 0.711 vs. 0.705) [40], Konik (our 0.703 vs. 0.702 and 0.70) [39,40], Norwegian Fjord (our 0.665 vs. 0.709 and 0.692) [9,40], Gotland Pony (our 0.644 vs. 0.643) [41], Shetland Pony (our 0.703 vs. 0.682) [40], and Exmoor Pony (our 0.635 vs. 0.560) [42]; however, breed sample size and origin may contribute to diversity value fluctuations. Overall, both this and the previous study [9] observe for the ENH higher HO than HE. The excess of HO as compared to HE seen in this ENH population likely reflects past introgression that has been retained up to the present. The high within-breed autosomal STR diversity of the ENH, as observed in this and a previous study [9], suggests that this native pony, with a positively growing population size of ~2500 individuals [10], has had no serious bottlenecks or pressure of selective breeding and has likely maintained ancestral allelic richness. However, in order to determine the origin of this genetic diversity and dynamics in time, further studies are needed based on genome-wide SNP or whole genome (WG) sequence data from modern and historic horses from the region.

The observed genetic diversity poses a question about the ancestry of the ENH and its relatedness to other horse breeds. To disentangle whether the variation in the ENH is historic or the result of recent introgressions, we started with global analysis by building an RLM tree where the cohort of 52 modern breeds (including the Przewalski’s horse) clustered into two major clades: one with 25 Thoroughbred-related breeds and non-draught/pony breeds and the other with 26 European draught and pony breeds, including the ENH (Figure S1). Importantly, our results based on 15 STRs were consistent with a previous genome-wide SNP analysis of 38 breeds showing that relationships between breeds reflect geographic origins and breed histories and that the majority of European breeds fall into Thoroughbred, Iberian, British Draught and Northern European categories [43]. Targeted analysis of the draught/pony clade, separated the ENH from all draught breeds and clustered it with a small group of 8 Northern European ponies (Figure 1 and Figure 2). Further analysis of the draught/pony clade excluded the Przewalski’s horse as the outgroup since the Przewalski’s horse has little admixture with domestic horses [44] (Figure S4) as the groups diverged 38–72 thousand years before present [45]. Additionally, previous breed relationship analysis of Nordic pony breeds consistently included the Exmoor Pony [15,25]. As expected, the genetically closest breed to the ENH was the Finn Horse (Figure 2B and Figure 3A). This is consistent with a previous study [9] and reflects admixture due to neighboring geographic locations and the recent history of the ENH. For example, it is documented that when purposeful pure breeding of the ENH started in 1921, 13 Finn Horse stallions were used to improve the ENH during a period of 15 years [5]. This shared gene pool is well illustrated by population structure analysis where the ENH and Finn Horse cluster together at K = 8 (Figure 3A). Because the Finn Horse and Norwegian Fjord were the only breeds in Group A (Figure 1) that were shared between this and the previous study of the ENH [9], detailed comparison of the two datasets was limited. Overall, within-breed diversity statistics were consistent between our study and that of Sild and colleagues (2019) for the allelic richness of the ENH (our 6.600 vs. 6.40), Norwegian Fjord (our 6.067 vs. 5.42), and Finn Horse (our 6.400 vs. 6.44) [9]. Interestingly, the genetic distances between the ENH and Norwegian Fjord were similar (our 0.097 vs 0.091), while the distance between the ENH and Finn Horse differed more (our 0.058 vs 0.081) [9]. Differences were also in admixture values between the ENH and Finn Horse (our 1.59% vs. 2.8%) and ENH and Norwegian Fjord (our 1.51% vs. 3.5%) between the two studies. These minor discrepancies in diversity statistics may be influenced by the particular sample sets used, though the overall number of individuals representing the ENH (33 vs. 35), Finn Horse (25 vs. 34) and Norwegian Fjord (50 vs. 38) were comparable.

Geographic closeness and extensive historical interactions through trade and warfare between Estonia and Scandinavia, and during the Viking Age also with Iceland and Britain, explain the close relatedness of the ENH to the Norwegian Fjord, Icelandic Horse, Gotland, Exmoor and Shetland ponies, although the two British ponies are clearly more diverged from the ENH as evidenced by the longest branch lengths in the neighbor-network tree (Figure 2B). Results for the Gotland Pony were somewhat surprising because long branches in neighbor-network tree (Figure 2B) and clear clustering in structure analysis at all K-values (Figure 3A) suggest a lack of recent admixture and more divergence than anticipated from close geographic proximity and historic interactions between western Estonia and Gotland. The Gotland Pony has relatively low diversity and this could be a result of isolation which would impact genetic distance. It must be noted that this is the only study that has analyzed the genetic relationship between the ENH and the Gotland Pony and one of the few population studies including the Gotland Pony [41] altogether. Perhaps future studies with more individuals and WG sequence data will reveal more shared genetics from the past between the two ponies.

Surprisingly, earlier findings indicate that the ENH shares a similar gene pool with Yakutian and Mongolian horses [9]. Our initial analysis of a global cohort of 52 modern breeds also included the Mongolian Horse (Table S1), but we did not observe genetic affinity between the ENH and Mongolian Horse as reported by Sild and colleagues (2019). Instead, the Mongolian Horse clustered with the Kyrgyzstan Horse and two Zemaitukas breeds (Figure S1), though Reynold’s genetic distance [18] between the ENH and Mongolian Horse were similar: 0.078 in our study (Table S7) and 0.068 in the previous study [9]. Therefore, the discrepancy between the two studies is likely due to the greater number and diversity of breeds used here. Despite this, close clustering of the ENH with the Nordic ponies in this study (Figure 1 and Figure 2B) indirectly supported the proposed connection between the ENH and Mongolian and Yakutian horses [9]. This is because a number of earlier studies show that Northern European breeds, such as the Icelandic Horse, Exmoor Pony, Shetland Pony, Norwegian Fjord and Finn Horse, share more genetic similarity to indigenous Asian breeds (Yakutian and Mongolian horses) than to European breeds of Thoroughbred ancestry [43,46,47,48]. It is thought that the genetic contribution of geographically distant Yakutian and Mongolian horses preceded the establishment of breeds [9] and is connected to ancient human migration routes from Siberia to Eastern and Northeastern Europe [46,49,50]. 

Ancient human migration from East to West may also explain our most unexpected finding that after the Finn Horse, the next genetically closest breeds to the ENH were the Hucul and Konik (Figure 2B and Figure 3A)—geographically the most distant from the ENH among the 8 pony breeds in Group A (Figure 1). The Hucul originates from the Carpathian Mountains and has supposedly a mixed ancestry from Tartar, Oriental, Arabian, Turkish, Nordic and Przewalski’s horses [51], whereas the Konik is based on primitive breeds from Eastern Poland [39,51]. A common characteristic of the Hucul and Konik is that both are primitive breeds and thought to be closely related or even direct descendants of the now extinct forest horse, Tarpan [39,51,52]. If so, our findings provide the first genetic evidence for the alleged Tarpan ancestry of the Estonian Native Horse as well [5]. Furthermore, the genetic similarity of the ENH to the Hucul and Konik in this study, and to Yakut and Mongolian horses [9], collectively suggest that horses were not domesticated in the territory of present-day Estonia, but rather arrived there with human migration. This is supported by archeological findings showing that even though wild horses lived in this territory ~10,000–5000 years before present (BP), they went extinct, and domestic horses were brought to Estonia only at early Bronze Age some 3000 years BP [1,2,5]. Recent WG ancient DNA studies corroborate this showing that 2717 year-old ancient horse bones from Ridala (a village on a western island of Estonia) are genetically the closest to 3574 year-old horse bones from Garbovat (Romania), located next to Carpathian Mountains and not far from the main hub of horse domestication in the Pontic-Caspian steppe [48]. Additionally, patterns of genetic diversity within the domestic horse suggest that ancestors from the Pontic-Caspian region may have contributed to modern European breeds [53]. Because of the emerging evidence that the ENH may have close genetic affinity to the wild Tarpan or early horse domesticates, it would be necessary to include the ENH, Hucul and Konik in ongoing studies deciphering the history of horse domestication based on ancient and modern equine genomes [48,54].

Since the discovery of a limited number of variable sites in the male-specific region of the horse Y chromosome (MSY) [30,55,56], it has been established that due to artificial selection, MSY diversity has dramatically reduced in all modern horses over the past ~2000 years [54,57,58]. The majority of European breeds cluster within the Crown group with MSY HGs A-L-S-T and, in the context of this study, carry derived alleles for SNPs rAY and rAX. Nordic breeds (Icelandic Horse, Norwegian Fjord, Shetland Pony), on the other hand, have HGs I and N and carry rAY and rAX ancestral variants [30,55]. Unsurprisingly, the majority (12/14) of ENH stallions fell into the Crown group clustering with draught breeds in HGs Ad and Tu (Figure 4). This is consistent with the autosomal RLM tree where the ENH groups with draught breeds in Clade II (Figure S1), as well as with documented introgression of the ENH with the Hackney and Ardennes in the 19th century [5]. However, the most interesting and unexpected finding was that two ENH males fell outside both the Crown and Nordic groups, carrying a novel HG defined by ancestral rAY and derived rAX alleles (Figure 4). This suggests that the ENH has contributions from unique patrilines with an origin yet to be determined by MSY sequencing and genotyping from additional males representing all closely related pony breeds in Group A (Figure 1).

Over the years, the genetic relations and history of horse breeds has been extensively studied using variable sites in mtDNA [34,59]. However, it is now a general observation that modern horses have high mtDNA diversity and lack of phylogeographic mitochondrial structure, resulting in limited correspondence between mtDNA haplotypes, breeds and geography [7,59]. Studies of ancient genomes show that high mtDNA diversity was already present in Scythian horses some 2300 years ago [60] and perhaps even earlier, because mitochondrial Bayesian Skylines suggest horse demographic expansion about 4500 years ago [48,61]. Nevertheless, and because ENH mtDNA has not been included in any of the previous global studies [34,59], determining ENH mitochondrial haplotypes in this study was a needed addition to autosomal and MSY data. As expected, even the small number of ENH horses studied (N = 9) separated into 5 haplogroups (Figure 5), indicating the presence of diverse maternal origins. In line with the genealogies based on autosomal STRs and MSY, mtDNA haplogroups related the ENH to draught and Pontic-Caspian breeds, including the Hucul (HGs C, P, M), to Nordic breeds (HG D), and to European breeds (HG L) [34,59,61]. 

In addition to the genetic makeup based on autosomal and haploid variants, the ENH was studied for the “gait” mutation—a single-base (C > A) substitution and nonsense mutation in the *DMRT3* gene, which is associated with “gaited” phenotype in homozygous (A/A) individuals [37]. “Gait” genotyping was undertaken to attest the anecdotal claim that the ENH is a “gaited” breed, but also because among the closely related pony breeds in Group A (Figure 1 and Figure 2B), the Icelandic Horse is a well-known “gaited” breed and selected for the phenotype since the Viking Age [37]. The mutation is present at low frequencies also in the Finn Horse [36] and the Hucul (Table 2; this study), but not in the ENH and Exmoor Pony (this study) or the Norwegian Fjord, Shetland Pony, Gotland Pony and Konik [35]. This suggests that with the exception of the Icelandic Horse [37], there has been no selection for gaited phenotypes in other breeds of this group. The observed low frequency of the mutation in the Hucul and Finn Horse may reflect natural diversity that never underwent selection, one way or another, or it may be the result of historic introgression. Nevertheless, genetic closeness of the ENH with both the Finn Horse and the Hucul encourages *DMRT3* genotyping in the entire population of ~2500 ENHs. It is possible that the anecdotal claim has some grounds and there are individuals carrying the “gait” mutation also among the ENH.

Finally, compelling evidence for the within-breed genetic diversity is the well documented richness of the ENH coat color patterns [12,13]. In addition to the common colors observed in many other breeds as the result of selective breeding and introgression, the ENH also shows the wild-type Dun coloration with primitive markings [12], such as seen in the Przewalski’s Horse and donkeys [62]. It is noteworthy that 19 of the 33 ENHs included in this study were Dun and contributed to previous research that resulted in the discovery of the *TBX3* mutations that underlie non-Dun coloration [14]. Strikingly, 13 of the 19 Dun ENHs carried a rare Dun genotype (A/G) that has only been identified in ancient pre-domestication horses [14] and in 4% of Koniks [63]. The fact that Dun coloration is documented in the Norwegian Fjord, Icelandic Horse, Shetland Pony and Hucul, but none of these breeds carry the rare and old A/G genotype [14,64], further highlights the ancient and distinct genetics of the Estonian Native Horse.

## 5. Conclusions and Future Approaches

Our analysis demonstrated that the Estonian Native Horse is a genetically distinct and diverse breed of ancient origin with no notable pressure of selective breeding. This is illustrated by the highest genetic diversity among the 8 genetically closest Northern European pony breeds, the presence of a unique MSY haplotype, and shared gene pools with primitive Eastern European horses like the Hucul and Konik. These findings, along with the knowledge that the breed has been managed semi-ferally with limited human interference [5], suggest that the genome of the ENH has been shaped predominantly by natural selection and may harbor genetic “jewels” lost in most high-maintenance domestic horse breeds. Therefore, the ENH requires more comprehensive analysis through whole genome sequencing to identify signatures of natural and human selection and sequence variants which underlie the breed’s adaptive capabilities. Special attention should be paid to the genetic features of the major histocompatibility complex and immunity-related genes because the breed is also known for disease resistance and excellent adaptation to harsh northern climate and nutritionally poor pastures. Small, local horse populations like the Estonian Native Horse are potential sources of unique alleles lost in commercial breeds, and appropriate conservation and breed maintenance strategies remain important to prevent further genetic erosion of this unique ancestral legacy.

## Figures and Tables

**Table 1:** Genetic diversity within 9 Northern Pony breeds (see Figure S1, clade III) based on 15 short tandem repeats (STR) markers.

Population (Abbreviation)	N	MNA (SE)	Ae (SE)	HO (SE)	HE (SE)	F_IS_ (SE)
Estonian Native Horse (ENH)	33	6.600 (0.423)	3.983 (0.309)	0.766 (0.033)	0.723 (0.025)	−0.059 (0.023)
Hucul (HU)	45	6.933 (0.441)	4.033 (0.418)	0.732 (0.047)	0.709 (0.037)	−0.018 (0.031)
Konik/Polish Primitive (PO)	53	7.133 (0.446)	3.820 (0.280)	0.696 (0.039)	0.703 (0.038)	0.006 (0.019)
Finn Horse (FH)	25	6.400 (0.567)	4.160 (0.367)	0.717 (0.056)	0.705 (0.053)	−0.019 (0.023)
Gotland Pony (GT)	43	5.533 (0.322)	3.024 (0.216)	0.650 (0.03)	0.644 (0.026)	−0.009 (0.024)
Icelandic Horse (IC)	38	6.067 (0.384)	3.526 (0.260)	0.711 (0.036)	0.686 (0.032)	−0.037 (0.024)
Norwegian Fjord (FJ)	50	5.867 (0.496)	3.355 (0.232)	0.694 (0.049)	0.665 (0.043)	−0.042 (0.019)
Shetland Pony (SP)	66	6.933 (0.358)	3.637 (0.249)	0.649 (0.037)	0.703 (0.024)	0.083 (0.032)
Exmoor Pony (EX)	70	5.133 (0.413)	2.984 (0.221)	0.619 (0.038)	0.635 (0.030)	0.035 (0.026)

N: sample size; MNA: average number of alleles per loci per population; Ae: average number of effective alleles per locus per population; HO: observed heterozygosity; HE: expected heterozygosity; F_IS_: inbreeding coefficient; SE: standard error.

**Table 2:** *DMRT3* gait mutation genotypes in Estonian Native Horses and Hucul and Exmoor ponies.

Breed	N	Wild-Type (C/C)	Carrier (C/A)	Mutant (A/A)
Estonian Native Horse	33	33	-	-
Hucul Pony	52	50	2	-
Exmoor Pony	29	29	-	-

## Supplementary Materials

The following are available online at https://www.mdpi.com/2073-4425/10/8/629/s1, Figure S1: Consensus RML Tree of 52 worldwide breeds with the ENH (boxed) using the Przewalski‘s Horse as an outgroup. Values at branch junctions correspond to bootstrap values as percentages (%)., Figure S2: Biplot representation of the FCA analysis in Figure 1 between the ENH (circled) and 22 European breeds. (A) Axes one versus two. (B) Axes one versus three, Figure S3: Line graph represents the true number of clusters, or populations (K), possible for Group A against DeltaK. Possible K value determined by number of populations plus one. DeltaK value based on the rate of change between the “log probability of data” (LnPD) in 10 iterations of each K value (Evanno et al., 2005). STRUCTURE estimated a number of 8 (K=8) discrete populations in groups A based on the amount of admixture patterns and is indicated with the highest data point (arrow), Figure S4: FCA plot for 52 global breeds including the Przewalski’s horse. (A) 3D FCA plot; (B) axis 1 vs axis 2, and (C) axis 1 vs axis 3, Table S1: Summary information about the breeds, number of individuals and population genetic analyses used in this study. For each breed there are shown the Clade designation in RLM tree, the group by FCA analysis, and HO, HE and Fis values with standard error, Table S2: Features of 18 MSY SNP markers including ID, indicative haplotype, ancestral allele, derived allele and flanking sequence, Table S3: List of breeds and individuals (with NCBI accession ID) used for mtDNA analysis, Table S4: Percentages of breed contribution to each STRUCTURE cluster for Group A, Table S5: MSY genotypes and haplogroups determined in ENH. Supplementary Table S6: mtDNA test statistics, Table S7: Reynold’s genetic distances for 52 global horse breeds.
