# Supplementary figures and images for: Population Genetic Analysis of the Estonian Native Horse Suggests Diverse and Distinct Genetics, Ancient Origin and Contribution from Unique Patrilines
Source: Genes (Basel). 2019 Aug 20;10(8):629. doi: 10.3390/genes10080629 (PMC6722507; doi:10.3390/genes10080629)

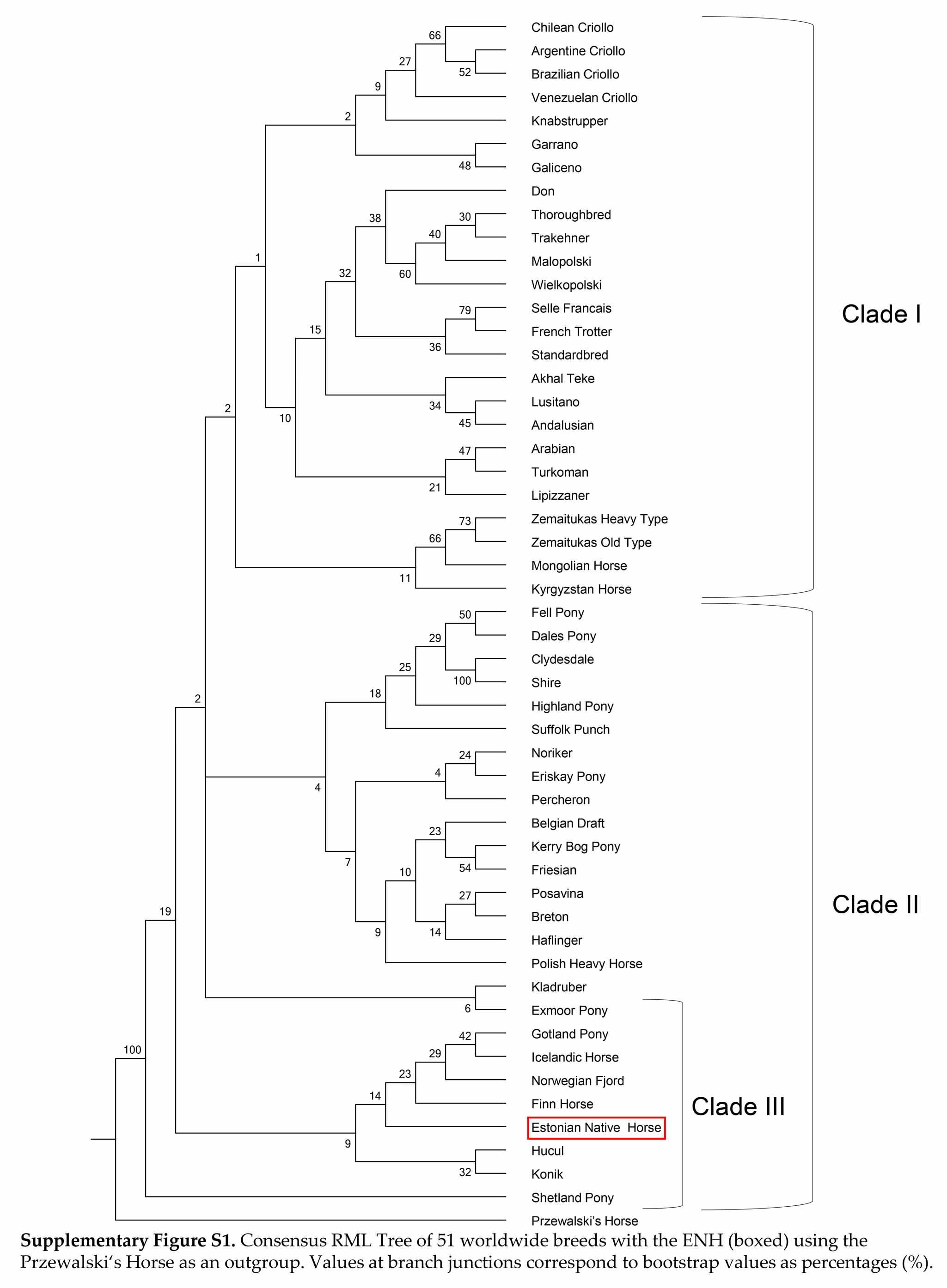

Supplement: Supplementary file 1 [file genes-10-00629-s001.zip › Supplementary Files/Supplementary Figure S1.jpg]

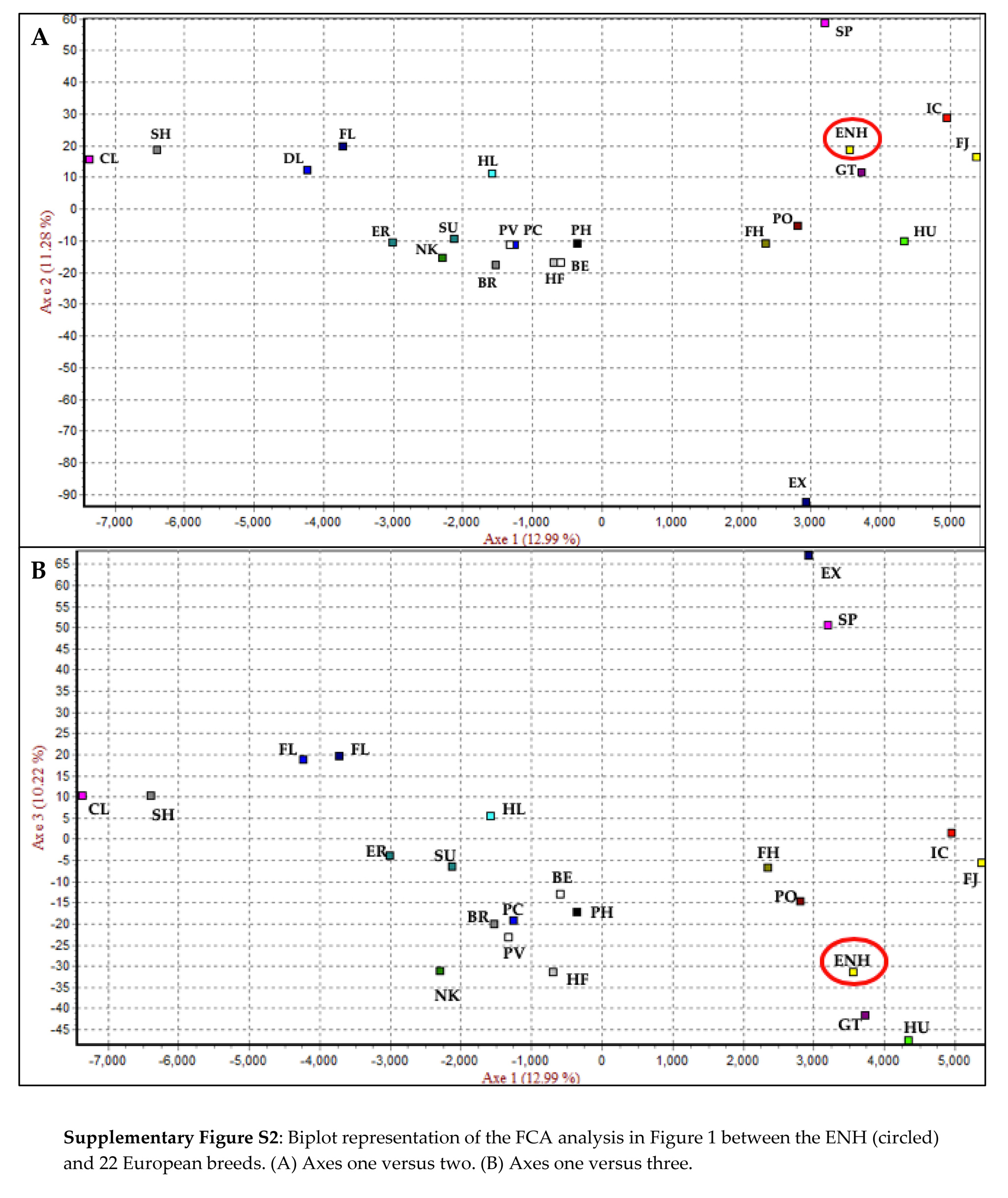

Supplement: Supplementary file 1 [file genes-10-00629-s001.zip › Supplementary Files/Supplementary Figure S2.jpg]

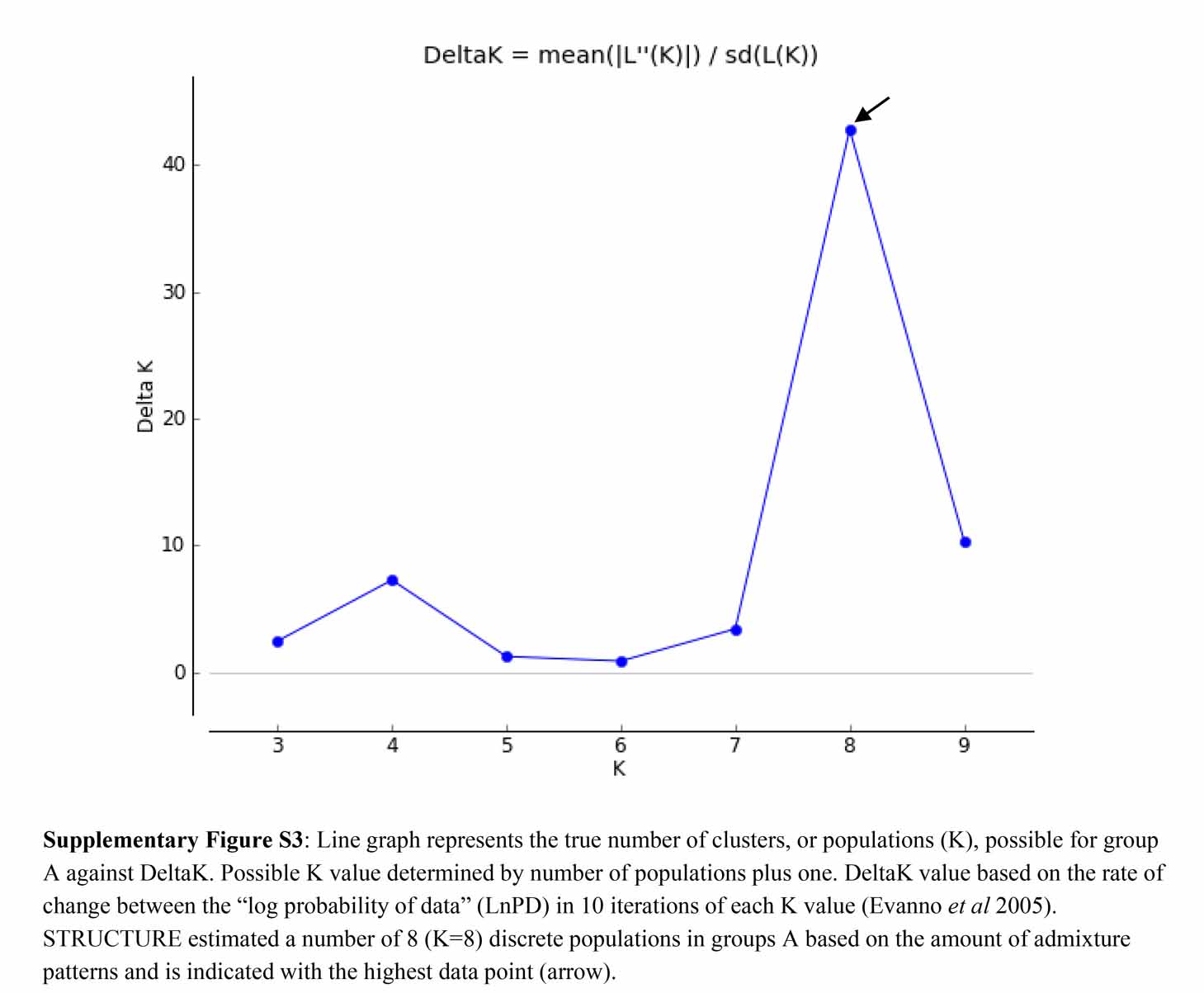

Supplement: Supplementary file 1 [file genes-10-00629-s001.zip › Supplementary Files/Supplementary Figure S3.jpg]

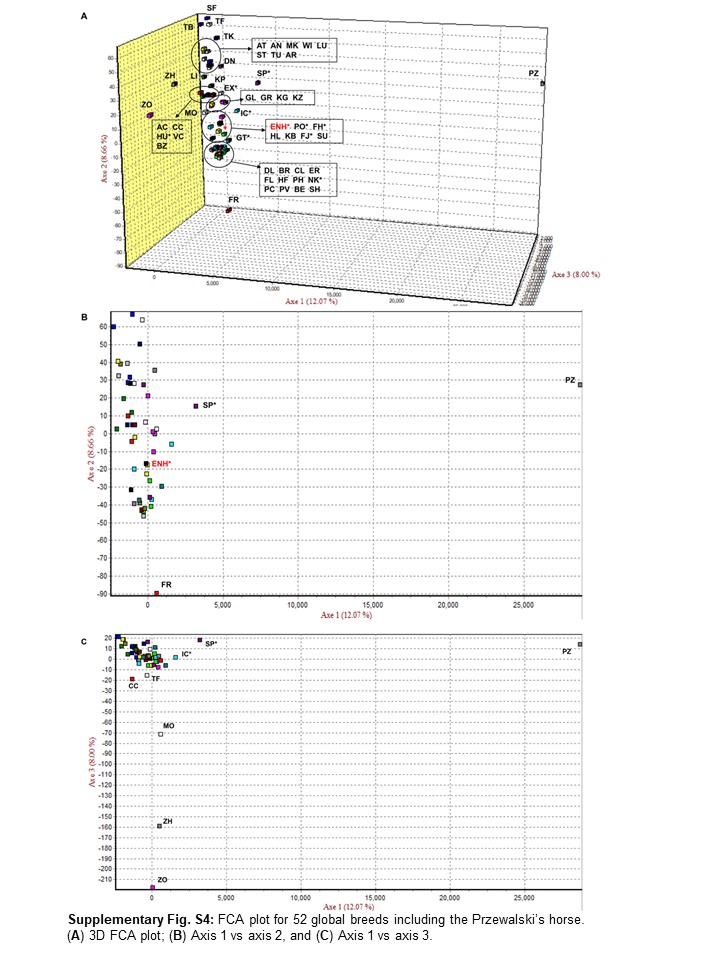

Supplement: Supplementary file 1 [file genes-10-00629-s001.zip › Supplementary Files/Supplementary Figure S4new.jpg]
